# Black bronchoscopy in a patient with pulmonary malignant melanoma: A case report

**DOI:** 10.1111/1759-7714.13571

**Published:** 2020-08-05

**Authors:** Hiroshi Sugimoto, Kyosuke Nakata

**Affiliations:** ^1^ Department of Respiratory Medicine Kobe Red Cross Hospital Kobe Japan

**Keywords:** Bronchoscopy, lung neoplasms, melanoma

## Abstract

A 47‐year‐old Japanese man was referred to our hospital with a one‐week history of chest discomfort. Chest computed tomography (CT) revealed a mass in the right upper lobe suspected to be primary lung cancer. A biopsy of the mass using endobronchial ultrasonography (EBUS) with guide sheath (GS) was performed, and a black‐colored mass was observed which occluded almost all of the right B^2^b bronchus. Immunohistochemistry of lung specimens was compatible with a diagnosis of malignant melanoma which was confirmed to be BRAF wild‐type. Furthermore, positron emission tomography (PET) and contrast‐enhanced magnetic resonance imaging (MRI) of the head revealed multiple metastatic lesions in the brain, liver, and bones. The patient was referred to another hospital for specific treatment. After that, the patient's melanoma was confirmed to have the BRAF wild‐type gene and PD‐L1 expression was 80%. Then, combined therapy of nivolumab plus ipilimumab was subsequently administered.

## Graphical abstract

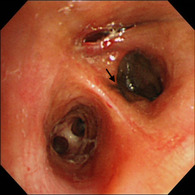

Black pigmentation of the airways (black bronchoscopy) can be caused by many etiologies. We herein reported a case of black bronchoscopy for pulmonary malignant melanoma.

## Introduction

**Figure 1:**
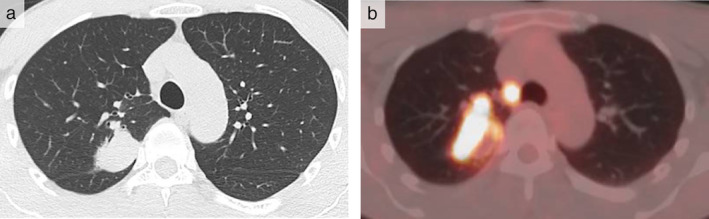
(**a**) Chest computed tomography (CT) scan showed a mass in the right upper lobe. (**b**) Positron emission tomography (PET) scan revealed a lung mass with a maximum standardized uptake value of 10.69.

**Figure 2:**
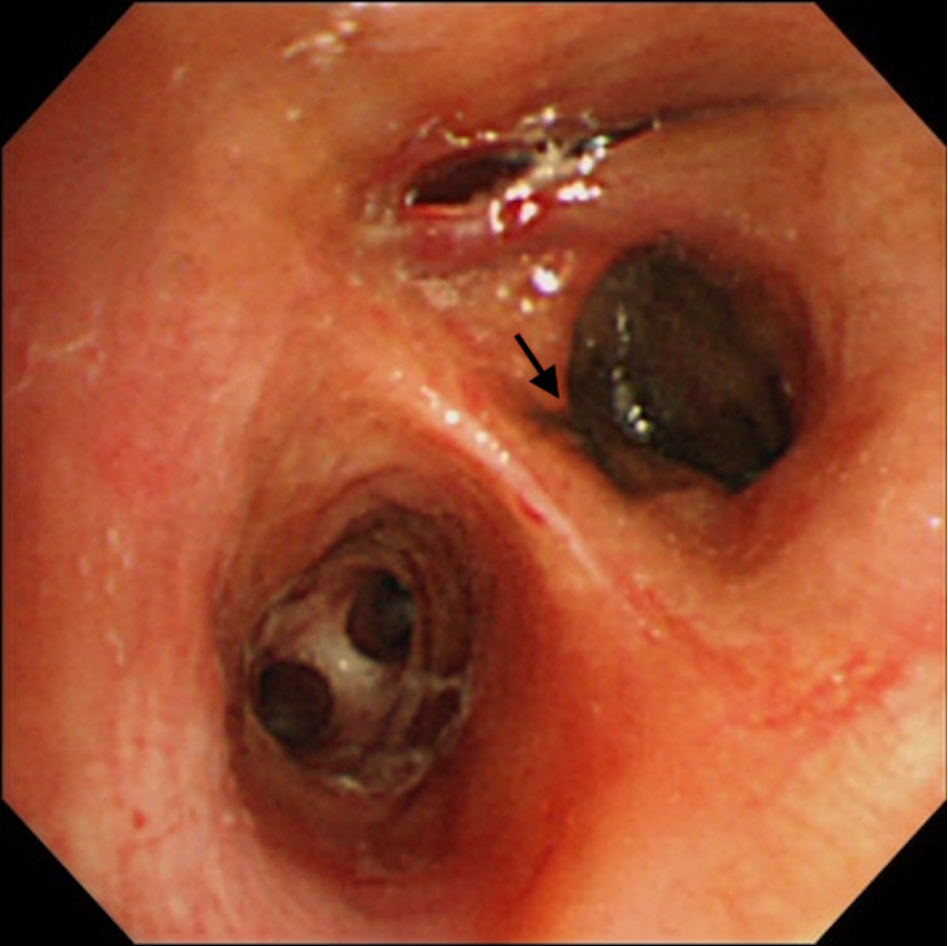
Bronchoscopy showed a black‐colored mass which was nearly occluding the right anterior B^2^b bronchus (arrow).

A 47‐year‐old Japanese man was referred to our hospital with a one‐week history of chest discomfort. He had never smoked and had no notable medical history. His vital signs were within normal ranges, and physical examination revealed no abnormalities, including dermatological changes. Chest computed tomography (CT) revealed a mass in the right upper lobe (Fig 1a) which was suspected to be primary lung cancer. A biopsy of the mass using endobronchial ultrasonography (EBUS) with a guide sheath (GS) was performed, and a black‐colored mass was observed which was seen to be occluding nearly all the right B^2^b bronchus (Fig 2). Immunohistochemistry of lung specimens revealed melanin pigmentation and malignant cells that were positive for HMB‐45, Melan‐A, SOX‐10, and vimentin and negative for S‐100, CKAE1/AE3, CK5/6, TTF‐1, CD‐45, and synaptophysin. These results were compatible with a diagnosis of malignant melanoma. Furthermore, positron emission tomography (PET) and contrast‐enhanced magnetic resonance imaging (MRI) of the head revealed the lung mass had a maximum standardized uptake value of 10.69 (Fig 1b) and there were multiple metastatic lesions in the brain, liver, and bones. The patient was referred to another hospital for specific treatment. After that, the patient's melanoma was confirmed to have the BRAF wild‐type gene and PD‐L1 expression was 80%. Then, combined therapy of nivolumab plus ipilimumab was subsequently administered. Written consent was obtained from the patient to publish the results of this study.

## Discussion

Black pigmentation of the airways (black bronchoscopy), as observed in our patient, may be caused by many etiologies such as deposition of coal or silver dust, hemochromatosis, amiodarone toxicity, alkaptonuria, and primary or metastatic malignant melanoma.^1^ There are no specific bronchoscopic findings that can confirm a diagnosis; thus, a diagnosis must be made comprehensively based on the clinical history and other laboratory and histological findings.^1^

Malignant melanoma is a cancer derived from melanocytes, usually in the skin, whereas noncutaneous primary lesions such as in the oral cavity and esophagus have also been reported.^2^ However, primary pulmonary malignant melanoma is very rare (0.01% of all lung tumors and 0.4% or less of all malignant melanomas).^2^ Although the pathogenesis of primary pulmonary malignant melanoma is not fully understood, migration of melanocytes to the pulmonary tissues during embryogenesis is considered to be the origin.^2^ It is difficult to distinguish primary lung cancer and melanoma by computed tomography, whereas black bronchoscopy can be helpful in the diagnosis of melanoma. It should also be noted that malignant melanoma can be amelanotic regardless of whether it is a primary or metastatic lesion.^1^

## Disclosure

All the authors declare that there are no conflicts of interest.
